# Supplementary material for: Attention to audiovisual speech shapes neural processing through feedback-feedforward loops between different nodes of the speech network
Source: PLoS Biol. 2024 Mar 11;22(3):e3002534. doi: 10.1371/journal.pbio.3002534 (PMC10957087; doi:10.1371/journal.pbio.3002534)
Supplement: S1 Text — (DOCX) [file pbio.3002534.s006.docx]

## Supplementary Text

## Supplementary speech envelope reconstruction and behavioural performance results.

## Linear mixed models (random intercept) and repeated factors Attention, Semantic Coherence, Auditory Quality and Visual Quality (random slopes for all repeated effects) were performed on SER accuracies separately for the dialogue speech stream and the background speech stream correlations.

## As expected, speech envelope reconstruction (SER) accuracy for the dialogue stream was significantly modulated by attention *(F_1,18.7_* = 67.2, *p* < .001, *η^2^* = .78) because SER accuracy was stronger when participants attended to the dialogue speech streams (mean *Δr* = .14, SEM = .004) than when they ignored the dialogue speech stream (*Δr* = .05, SEM = .005). There were no significant effects of attention on the SER accuracy for the background speech stream.

## There were significant two-way interactions: between Attention and Semantic Coherence (F_1,23.5_ = 7.4, *p <* .01, *η^2^* = .24 ) because, when the participants attended to the dialogue speech stream, SER accuracies were stronger for semantically coherent dialogues than incoherent dialogues; and between attention and auditory quality (*F_1,21.6_* = 5.4, *p <* .03, *η^2^* = .20) because, when participants attended to the dialogue speech, SER accuracies were stronger for dialogues with good auditory quality than dialogues with poor auditory quality. There was also a significant four-way interaction between all factors (*F_1,24,.6_ =* 5.8, *p <* .02, *η^2^* = .19; see S1 Fig).

## For the background speech stream, there was a significant main effect of Auditory Quality (*F_1,24.6_ =* 5.6, *p <* .03, *η^2^* = .19), because SER accuracies were stronger when the dialogue stream was presented with good (*Δr* = .03, SEM = .004) than poor auditory quality ( *Δr* = .009, SEM = .004); and a significant main-effect of Visual Quality (*F_1,26.9_ =* 7.6, *p <* .01, *η^2^* = .22), because SER-correlations were stronger when the dialogue speech stream was presented with poor quality( *Δr* = .03, SEM = .004) than good quality (*Δr* = .007, SEM = .004).

## Analysis of the performance in the EEG experiment for the attend speech task yielded partially similar results as the SER analyses. There were significant main effects of Semantic Coherence (*F_1,18_* = 109.0, *p <* .001, *η^2^* = .85), Auditory Quality (*F_1,18_ =* 30.6, *p <* .001, *η^2^* = .63) and Visual quality (*F_1,18_ =* 28.6, *p <* .001, *η^2^* = .61). All effects were due to performance being better with coherent and good quality dialogues (see S2 Fig left). There was also a significant three-way interaction between the factors (*F_1,18_* = 4.6, *p <* .04, *η^2^* = .2). However, a comparison of S1 and S2 Figs reveals that this interaction arose due to different effects in the behavioural performance compared to the SER-correlations. There were no significant differences in performance between the coherence and quality conditions during the ignore speech task (S2 Fig middle).
