# Supplementary material for: Attention to audiovisual speech shapes neural processing through feedback-feedforward loops between different nodes of the speech network
Source: PLoS Biol. 2024 Mar 11;22(3):e3002534. doi: 10.1371/journal.pbio.3002534 (PMC10957087; doi:10.1371/journal.pbio.3002534)
Supplement: Attachment — Submitted filename: wikman_et_al_2023_response_letter_PlosBiology.docx [file pbio.3002534.s011.docx]

**Dear** **Professor Schnell,**

Thank you for giving us the opportunity to revise our manuscript *Selective attention to audiovisual speech routes activity through recurrent feedback-feedforward loops between different nodes of the speech network* based on the comments from the reviewers. We believe that in the attached revised manuscript we have adequately taken into account the reviewers’ constructive comments and helpful suggestions and that the manuscript has consequently improved. Please, find below the reviewers’ comments and our replies to them and explanations of revisions prompted by the comments. Additional small spelling mistakes were corrected, and the corrections are highlighted in red. We hope that you will find our revised manuscript publishable in *PLOS Biology*.

Sincerely,

Patrik Wikman, PhD

**Reviewer comments**

**Reviewer 1:**

**Genneral assessment:** The present study combined electrophysiology and functional brain imaging to explore the cortical regions involved in top-down modulations during multi-talker situations. The task design very ambitiously aimed to simultaneously manipulate the focus of attention, semantic coherence (context), and attentional demand (auditory and visual quality). They demonstrated neural tracking of attended speech supported by the ventral auditory stream. Furthermore, the attentional effect on the accuracy of speech envelope reconstruction depended on the course of the dialogue, suggesting that predictive and attentional mechanisms interact via neural tracking.

Overall, I support the publication of this study, with a few suggestions.

**Response:** We thank the reviewer for the supportive comments. Please, find our detailed response to the comments below.

**Comment 1.1**: The paragraph explaining the experimental design in lines 89-99 needs to mention the hypothesis or describe the rationale behind using each type of experimental manipulation. It is necessary to include a reason and related hypothesis for using semantically coherent or incoherent dialogues. The introduction could benefit from explaining this.

**Response:** Thank you for pointing this out. The audiovisual and semantic manipulations were used because they significantly interacted with attention in our previous fMRI study (Wikman et al., 2020), and thus we wanted to utilise exactly the same paradigm in the present EEG-FMRI fusion analysis. We agree that the factorial design is fairly complex. However, to perform representational similarity analysis (RSA), it is advisable to have enough nodes in the representational similarity matrix to find the multidimensional correspondences between different types of measures (here EEG and fMRI data and models; see Kriegeskorte et al, 2013). Additionally, semantic, and audiovisual quality has been reported to influence SER in previous studies (Broderick et al., 2019; Rimmele et al., 2015; Haider et al., 2022). But because attentional allocation has not always been considered in these studies, we wanted to study whether these manipulations influence speech envelope reconstruction (SER) only when the speech is attended.

We originally thought that for the sake of brevity, paragraphs in the Results section referring to expected results would suffice in grasping the hypotheses of the study. We now, however, added the expected results in the second last paragraph of the Introduction, which we agree makes the study focus clearer.

Line 99–107:

*We expected that attending to the dialogues would increase speech envelope reconstruction (SER) accuracy of the dialogue stream and enhance related early and late neural temporal response components, with opposite effects for the background speech stream. Based on results from our previous fMRI study (Wikman et al., 2020), attentional modulation of the dialogue stream SER accuracy was expected to be temporally variable, changing from line-to-line in a nonlinear fashion. Additionally, we expected SER accuracy to be greatest for dialogues with good audiovisual quality (Rimmele et al., 2015; Haider et al., 2022) and coherent semantics (Broderick et al., 2019). However, we also wanted to determine whether this applies only to attended speech.*

**Comment 1.2:** Please briefly define the term representational dissimilarity matrix at the first mention in the text.

**Response:** We added the following paragraph:

Line 345–348:

*A representational Dissimilarity Matrix (RDM) is a geometrical description of the data, showing an assembly of all pairwise dissimilarities across neural responses or model predictions to different stimuli or experimental conditions (Kriegeskorte et al, 2013).*

**Comment 1.3**: Factorial design means that all subjects performed the two tasks (selective attention) under all types of semantic coherence and attention demand conditions. Is that correct? Please disambiguate in the text.

**Response:** Yes, this is correct, we added the following sentence:

Line 144–147:

*We employed a 2 × 2 × 2 × 2* *within-subjects factorial design, where participants performed three runs including all possible combinations of the Attentional Task (attend, ignore), Auditory Quality (good, poor), Visual Quality (good, poor) and Semantic Coherence (coherent, incoherent).*

**Comment 1.4):** Was there enough statistical power to test all the effects of attention demand, selective attention, and context?

**Response:** Please see also our response to Comment 1.3. We used a within-subjects design and thus all main effects and interactions are tested against their own error term and are orthogonal to each other. This provides a considerable advantage in statistical power as compared to between-subjects designs.

Including all four factors resulted in limitations on the number of repetitions of each condition for any given subject. The current study included one dialogue/condition/run of the experiment, which could be considered little. However, one needs to bear in mind that this means approximately 2–3 minutes of continuous speech per condition across all three runs (the dialogues were, on average, 1 minute in duration with breaks between the lines). Further, using TRFs and SER instead of standard ERPs greatly increases statistical power, as one can use the whole speech signal to estimate the neural response functions.

However, as we wanted to produce robust estimates, we chose to study the effect of line number and segment only for a combination of the Attentional Task factor (see lines 180–243) and sound stream (dialogue, background), instead of all possible interactions with the other factors (we also had no hypotheses regarding these other factors).

We had the statistical power to show an effect of attention on both SER for the dialogue stream and TRFs for both the dialogue and the background stream. In the SER analyses we also showed interactions between Attentional Task and the other factors (Semantic Coherence, and Audiovisual Quality) for the dialogue stream, which we report in the supplement:

Supplement:

*There were significant two-way interactions: between Attention and Semantic Coherence (F_1,23.5_ = 7.4, p < .01, η^2^ = .24 ), because when the participants attended to the dialogue speech stream, SER accuracies were stronger for semantically coherent dialogues than incoherent dialogues; and between attention and auditory quality (F_1,21.6_ = 5.4, p < .03, η^2^ = .20), because when participants attended to the dialogue speech, SER accuracies were stronger for dialogues with good auditory quality than dialogues with poor auditory quality. There was also a significant four-way interaction between all factors (F_1,24,.6_ = 5.8, p < .02, η^2^ = .19; see Suppl. Fig. 1).*

Thus, we found no indication of a lack of power. We now specify in the main text that effects for Semantic Coherence, Auditory and Visual Quality can be found in the Supplement:

Line 175–178:

*Please refer to Suppl. Text and Suppl. Fig. 1–2 for all other significant effects in these linear mixed models and their correspondences to the behavioural performance results (e.g., effects related to Semantic Coherence, Auditory and Visual Quality).*

**Comment 1.5:** Typically, neural tracking is used to compare the effect of attention on the attended versus background stream. Still, the present design allows the authors to compare the task effect of selective attention on ignored versus attended speech. Was the same sensory input compared in two task conditions, with the same dialogue presented in different conditions? Is there a confound of repetition?

**Response:** It is true that neural tracking is usually used to compare the effect of attention on the attended versus the background stream. However, in our case the attended stream was always the dialogue stream, which was audiovisual and spoken by two speakers, while the background stream was audio-only and always spoken by the same (third) speaker.

Thus, the confound of audiovisuality and speaker would always be present in comparisons between the dialogue stream and the background stream (as can be seen in Fig. 3, the two streams yield also strikingly dissimilar TRFs irrespective of attention).

Therefore, we chose not to generally do comparisons between the dialogue and background streams. Instead, our attention manipulation stemmed from the two tasks: 1) the attend speech task, where the participants actively processed the visible dialogue, and 2) the ignore speech task where the participants ignored all speech stimuli and performed a visual task.

As we state on line 97–99, this also enabled us to study the effect of selective attention on both the dialogue stream and the background stream separately. This has not to our knowledge been studied before.

The only analyses where the dialogue streams and background streams are included in the same analyses is the line number and segment analyses (see also our response to comment 1.7), because here we were not interested in the effect of attention, per se, but whether temporal effects differ based on attentional allocation and speech stream. As reported on line 192–211, temporal effects were only present for the attended dialogue stream.

Lastly, there is no repetition confound, as each dialogue and background speech segment was unique (i.e., no speech segment was repeated for any participant). Furthermore, we controlled order and stimulus related effects as is stated on Line 713–716:

*Within the functional runs, the attend speech task and the ignore speech task were presented in an alternating order. The order of conditions and dialogues presented was pseudorandomized. Because we could not entirely randomise the videos into the 16 conditions per run, we used the Latin square to construct four different versions of the experiment (see Suppl. Table 3 in Wikman et al., 2020).*

However, since the manuscript starts with the Results, we now also explicitly make these points clear also in the beginning of the results section. First, we bring up that stimulus related effects and order effects were controlled. Second, we bring up why we did not compare the dialogue stream to the background stream:

Line 147–153:

*To control for stimulus effects, each dialogue/background speech segment (across all conditions and runs) was unique (i.e., each was heard only once). The condition order and the dialogue allocated to each condition varied between participants (see “Procedure”). Because the dialogue stream comprised audiovisual speech, while the background speech comprised purely auditory speech, spoken by a speaker different from the ones having the dialogue, the main comparisons were conducted separately within speech streams.*

In addition, we added to the discussion a paragraph to state that manipulations and comparisons of the two streams would be a promising direction in future studies.

Line 464–467:

*As the background speech is affected by manipulations of the attended speech, future studies could do manipulations for both sound streams (dialogue and background streams) and alternate the focus of attention between the two streams.*

**Comment 1.6:** Was there a significant increase in neural tracking of the speech from attended dialogue compared to the background stream?

**Response:** Please, see our response to comment 1.5: We generally did not compare the tracking of these two streams because of the confounds (audiovisual vs. visual, different voices) complicating interpretation of such comparisons. As can be seen in the revised Fig. 3, the background stream yielded smaller TRF amplitudes than the dialogue stream irrespective of attention, and the mean SER accuracy was clearly smaller for the background speech stream than for the dialogue stream, more so during the attend speech (i.e., attend dialogue) task. See below for the mean SER and standard error of mean (in brackets) separately for the two streams and the two task manipulations:

| Task | Attend Speech | Ignore speech |
| --- | --- | --- |
| Dialogue stream | .136 (.004) | .059 (.005) |
| Background stream | .019 (.007) | .015 (.005) |

We noted a rounding error for SER in the ignore speech task for the dialogue stream and corrected it from .05 to .06 on line 172.

**Comment 1.7:** Lines from 164: "First, we examined within-line effects, that is, whether SER accuracy changed within the line (lines were divided into four equal length segments)"; "Line of the speech stream (1-7), and Segment of the line (1-4)". Regardless of which line it was, the segment order affected the accuracy of the SER. Is that correct? Why was segment order analyzed instead of the effect of the line? Why do the authors assume that SER accuracy changes during the dialogue are more representative through the segment than the lines?

**Response:** We did study both whether SER changed as a function of line number and within the line. As we now clarified in the Introduction (see our response to comment 1.1), in our previous fMRI study we found that attentional modulation of speech changed in a nonlinear fashion from line-to-line of the dialogue. However, the temporal resolution of fMRI did not allow us to examine whether this effect arose due to differential processing of the dialogue speech stream or the background speech stream. Further, the temporal resolution of fMRI did not allow us to evaluate whether attentional effects changed as the line proceeded (which we now explicitly state in the text, see below) in our previous study. Therefore, because we used EEG and SER in the present study, we chose to evaluate whether SER accuracy shows similar nonlinear temporal effects as was reported between lines in our previous fMRI study within each line (i.e., the segment analysis).

We want to highlight that indeed both the within-line effects (segment-to-segment) and the line number (line-to-line) effects were significant. But unlike the line number effect, which was nonlinear, the within-line effect was linear (which we discuss extensively in the Discussion, lines 502–568).

We appreciate that the rationale of the text was unclear, and we did not spell out adequately what was assessed in the different analyses. Therefore, we changed the text as follows:

Line 180–211:

## *In our previous study utilising fMRI only, we reported that in the auditory cortex attention related modulations changed in a linear-quadratic fashion during the dialogue (i.e., increased in the beginning of the dialogue and abated thereafter; see Fig. 2c, Wikman et al., 2020). Neuronal tracking has been suggested to be most strongly affected by neuronal processes in the superior temporal cortex (O’sullivan et al., 2015), and thus we expected similar temporal effects here. However, unlike with fMRI, here we could assess whether the previously reported temporal modulations were due to the processing of the attended or the ignored speech stream because they are separable in the EEG data. Further, utilising the EEG data in the present study also allowed us to evaluate whether attentional modulation changes within each line in a similar fashion as between the lines, which was not possible in our previous study due to the temporal resolution of fMRI.*

## *Thus, first we examined within-line effects. That is, whether SER accuracy changed within the line (lines were divided into four equal length segments). As seen in Fig. 2b, there was a significant linear decrease in SER accuracy throughout the line for the dialogue stream when it was attended, and to some extent for the dialogue stream when it was ignored but not for any other combinations of Attentional Task and Speech Stream (Fig. 2b; significant Condition × Segment interaction, F_9,116_ = 11.2, p < .001, η^2^ = .46; linear mixed model with the repeated factors Condition (attend speech task dialogue stream; attend speech task background stream; ignore speech task dialogue stream; ignore speech task background stream) and Segment (1–4)).*

## *Next, we analysed whether attention changed SER accuracy from line-to-line in a similar non-linear fashion as previously seen in our fMRI study (see Wikman et al., 2020). As seen in Fig. 2d (left), SER accuracy showed a similar temporal profile changing from line-to-line as previously observed with fMRI. In other words, SER accuracy increased during the first lines of the dialogue and abated towards the end. Further, this temporal effect was only evident when the participants selectively attended to the dialogue stream (Fig. 2d left; significant Condition × Line Number interaction, F_18,137.3_ = 2.4, p < .002, η^2^ = .24; linear mixed model with the repeated factors Condition (attend speech task dialogue stream; attend speech task background stream; ignore speech task dialogue stream; ignore speech task background stream) and Line Number (1–7)).*

**Comment 1.8**: What does the timescale of the TRF response reflect? It is mentioned that it can be conceptualized as an ERP to a continuous variable, but what continuous variable the authors refer to needs to be clarified. Is it the speech signal?

**Response:** The timescale is similar to that of ERPs. The continuous variable is indeed the speech signal. Or more specifically it is the speech-amplitude-envelope signal, which is used as a continuous predictor.

Thus, if a basic ERP study with discrete short sound segments would be analysed using TRFs, a sound-amplitude envelope predictor would yield very similar results and similar time scales as a simple boxcar predictor, which again would be very similar to standard ERPs generated with the standard averaging method. Basically, the TRF gives the average transformation from the speech amplitude to the EEG signal. We added the following text:

Lines 280–284:

*With some caveats, TRFs can be conceptualised as event-related potentials (ERPs) to a continuous variable, which here is the continuous speech amplitude envelope, and where the timescale refers to time lags to the speech signal (see “First-level analysis of EEG data” Kaufman et al, 2022).*

**Comment 1.9:** In line 264, it is stated that selective attention enhanced the TRF for the dialogue stream, but it would be more precise to say what was compared here (the dialogue vs. background stream)

**Response:** Please see also our response to comment 1.5 and 1.6, the dialogue and background stream were never compared for the TRFs. We now added the following specification:

Line 292–296:

*Selective attention significantly enhanced the TRFs for the dialogue stream (i.e., there was a significant main effect of Attentional Task, i.e., attend speech > ignore speech) and this effect was present at two intervals between 0–800 ms, first at ca. 50–100 ms and then at ca. 200–400 ms after sound envelope changes (paired permutation t-tests, df = 18).*

**Comment 1.10:** It is hard to interpret the difference in conceptualization of the results for the attentional differences in TRF-RDM analysis and the one that compares TRF between ignored and attended speech. Please elaborate on it, at least in the discussion section.

**Response:** It was to be expected that the multivariate TRF-RDM analysis would yield different results than the univariate TRF comparisons. First, the univariate analysis focused of 8 frontocentral electrodes chosen to maximize auditory attentional effects, while the TRF-RDM analysis utilised all the EEG channels.

Further, RSA yields significant results both when there is a general difference in amplitudes between the conditions across channels and when the response pattern across channels is different between the two tasks. Accordingly, RSA is usually more sensitive but less specific than univariate analyses. Thus, the TRF comparison reveals the direction of difference, that is, was the signal larger/smaller due attentional manipulation, whereas TRF-RDM points to the differences in information between the two tasks.

We now clarified the in the Discussion text relating to this:

Line 471–476:

*RSA of the EEG data for both the dialogue stream and the background stream revealed that selective attention strongly modulated TRFs at several latencies that have not been reported in previous studies. Note that unlike the univariate TRF analyses the RSA analyses utilised all EEG-channels and were thus expected to find significant patterns in the channels outside those usually studied in auditory attention studies (e.g., frontocentral electrodes).*

**Comment 1.11:** In Fig. 4C, the fMRI contrast shows the most substantial contrast on the visual cortex. How did it come about? That is the source of the most prominent computational difference between the two tasks (visual vs. auditory). It suggests that this analysis only reflects the most cortical differences between the two tasks, but in line 372, the authors state that the analysis is not due to the main effect of the task. Thus, it needs to be discussed.

**Response:** A strong Attentional Task effect in the visual cortex was to be expected because the dialogues were audiovisual, and the participants attended both to the visual and the auditory speech of the two speakers. For a more in-depth discussion on the visual effects and the fMRI data, please refer to Wikman et al., 2020.

Indeed Fig. 4C shows where the Attentional Task main effect model is strongest. As stated on line 361–371, other areas than the ones depicted here also were associated with a main effect of Attentional Task. But to disambiguate which areas showed the strongest effects, we show only above average correlations.

The text on line 373–387 relates to the TRF-fMRI fusion results. For these analyses, we factored out the main effect of Attentional Task using partial correlation, because as can be seen in Fig. 4C, it occludes any subtle effects. That is, if Attentional Task would not have been factored out, the fusion analysis would have shown only the timepoints and regions when the main effect of Attentional Task was significant, which had been almost throughout the brain and throughout the TRF-response window.

Consequently, TRF-fMRI fusion does not tell us correspondences between fMRI-EEG main effects of Attentional Task, but rather correspondences between higher order-interactions between the Attentional Task and the semantic and quality manipulations. We now highlighted this in the text to make this clearer:

Line 381–387:

## *We also corrected for the main effect of Attentional Task in the TRF-fMRI fusion analysis because this effect was global in both the TRF and the fMRI responses (see above; Fig. 3b-c), and thus masks subtle differences between different regions. Thus, the main effect of Attentional Task (Fig. 3b-c) does not contribute to the TRF-fMRI fusion, and the correspondences arise instead due to other more complicated correspondences between the EEG and fMRI RDMs (see below).*

**Comment 1.12:** Correlation between TRF and fMRI RDM arose in 16 ms and 30-78 ms for the dialogue stream and about 8 ms for the background stream. This is contradicting, given that fMRI has a really low temporal resolution. What these timescales refer to needs to be defined more clearly (EEG TRF response?). Also, the fact that TRF and fMRI datasets were collected from unidentical subjects needs to be made explicit in the main text, not just in the methods section.

**Response:** It is important to note that all the timing information was obtained from the EEG-data and all the spatial information from the fMRI data. Thus, the timing resolution of fMRI did not factor in for this analysis. The effects at 8 and 16 ms means that the RDMs obtained from the TRFs at 8 and 16 ms correlate significantly with RDMs in the spatial locations in the fMRI data. Thank you for pointing out that we did not explicitly state that the fMRI data was from different participants. We clarified these two points on lines 398–399 and 361–363:

*Note that the timing information in this analysis is derived entirely from the TRF data and the spatial information from the fMRI data.*

*We also performed the same RSA analysis on our fMRI data that used the same paradigm but different participants (the same fMRI data was used for the TRF-fMRI fusion, see below).*

**Comment 1.13:** I suggest adding a reference that also shows the selective attention effect of unattended speech in a broad cortical network at lines 420, 426 and 436: Tóth, B., Farkas, D., Urbán, G., Szalárdy, O., Orosz, G., Hunyadi, L., ... & Winkler, I. (2019). Attention and speech-processing-related functional brain networks are activated in a multi-speaker environment. PLoS One, 14(2), e0212754.  (mi lendület cikkünk PLOS one )

**Response:** Thank you for this suggestion. We have added this reference in the suggested positions.


Minor comments

**Comment 1.14:** Fig. 2C shows the temporal changes in attentional modulation. It needs to be clarified what the color coding on the cortical surface represents. The label for the y-axis needs to be included in the diagram below, and the definition of primary speech network should be stated at least in the captions.

**Response:** Done as suggested

**Comment 1.15**: In line 191, the first time the slow temporal effect is mentioned, it would be helpful to define it (line effect - slow temporal effect).

**Response:** Done as suggested.

**Comment 1.16:** In Fig. 3A, the text above the TRF for each EEG channel (bottom panel) is too small to read.

**Response:** We have made the suggested font larger, as well as other fonts larger in Fig. 3.

**Reviewer 2:**

**General comment:** Wikman and colleagues report EEG and fMRI data from human participants selectively attending to audiovisual speech of two talkers (dialogue) with concurrent background speech (cocktail party situation). Participants either attended to the (foreground) speech (solving questions after every dialog) or attended to a visual cross (i.e. ignoring the speech and answering questions related to the turning of the cross). The quality of the sound and visual stimuli (speech and video of talkers) was manipulated (good vs. poor). The EEG results showed that attention increased the neural tracking of speech. Moreover, the accuracy of the speech envelope reconstruction was modulated over time of the dialogue. This was mirrored by the fMRI results. Applying temporal response function analysis to the EEG data (yielding temporal information similar to ERP analysis) revealed an effect of attending to the dialog in a window around 75 ms and another around 300 ms. Multivariate representational similarity analysis on the temporal response functions (yielding a pointwise temporal response function representational dissimilarity matrix along the time course of each stream) revealed an effect of the effect of attentional task (attend versus ignore the sounds). There was an effect for the dialogue but also for the background speech. The fMRI data revealed for this contrast effects in many (also non-auditory) regions of the brain. Finally, performing EEG-fMRI fusion revealed a series of back (to auditory) and forth (from auditory) correlations in a temporal window of few hundred of ms for the dialogue stream (first, at less than 20 ms after changes in the sound envelope in dorsolateral and dorsomedial frontal regions, which were succeeded by correlations in auditory regions, which were followed by correlations in anterior temporal areas, and then followed by correlations back to auditory and non-auditory regions). Also for the background stream
back-forth correlations were observed, however, starting at a latency of around 450 ms. While the EEG and fMRI analysis replicated (and partly extended) existing knowledge of attention effects in cocktail party situation, the EEG-fMRI fusion data hint at feedforward-feedback informational flow between auditory areas and areas associated with speech and executive functions.

In my opinion, this is a highly innovative study using a clever experimental design and a sophisticated analysis of the EEG and fMRI data. The majority of within auditory attention research and between audition versus vision attention research results were confined to either showing attention effects in time or in space. A chronometry of the brain's network of within and between modality auditory attention effects has (to my knowledge) never be performed. Thus the present results are a huge achievement in the classic but still very timely topic of auditory attention. The results are convincingly framed within current ideas about neural circuit learning / selection and recurrent low-level-sensory-high-level-non-sensory loop processing  (e.g. by Michael Kilgard and Erkki Schröger).

Though the manuscript Is well written and is in a very good shape, I have some comments:

**Response:** We want to thank the reviewer for taking the time and giving the valuable feedback, which we have integrated in our revision (see below).

**Comment 2.1:**
Discussion
As Wikman a colleagues somehow open a new avenue in auditory attention research, they might shortly set their findings into a somewhat broader context, that is, to different but still related research fields. I have three possible "candidates" in mind, but there might be more appropriate ones. In psychology Jones and Riess were forerunners in studying an conceptualizing the dynamics of attending to sound (though their dynamics relates to larger temporal scale and to the entrainment effects set by the context; this could be related to the dialogue lines findings), e.g. Large, EW; Jones, MR (1999): The dynamics of attending: How people track time-varying events. In: Psychological Review 106 (1), S. 119-159. DOI: 10.1037/0033-295X.106.1.119. In neurobiology, for example, Jonathan Fritz, Mounya Elhilali, and Shihab Shamma were pioneers in showing auditory attention effects on the respective receptive field can be rapidly adapt; this could be taken as a possible biological
implementation of some of the results; e.g., Fritz, JB; Elhilali, M, Shamma, SA. (2007): Adaptive changes in cortical receptive fields induced by attention to complex sounds. In: Journal of neurophysiology 98 (4), S. 2337-2346. DOI: 10.1152/jn.00552.2007. In computational modelling the auditory attention effect in cocktail party situations the EEG temporal response function approach has been modelled with recurrent neural network and reinforcement learning methods.; e.g. Geravanchizadeh, Masoud; Roushan, Hossein (2021): Dynamic selective auditory attention detection using RNN and reinforcement learning. In: Scientific reports 11 (1), S. 15497. DOI: 10.1038/s41598-021-94876-0.

**Response:** Thank you for pointing out this key work. We have now integrated it in the following sections of the Discussion:

Line 532–533

*Similar slow fluctuations of attentional effects have been previously described using fMRI (Wikman et al., 2020; Ylinen et al., 2022) and behavioural experiments (e.g., Large et al., 1999).*

Line 550–556:

*Several animal studies have shown that attentional tasks cause transient–persistent plastic changes in auditory neuronal response profiles (e.g., Fritz et al., 2007, 2005). The conundrum, however, has been that some studies have indicated that behavioural performance accuracy persist after the original plastic changes have subsided (Reed, et al., 2011). Therefore, Kilgard proposed that when the task is initially learned, all possible neuronal networks that may be useful to solve the task at hand are recruited.*

Line 581–584

*These findings may offer key insights in improving dynamic computational models of selective attention in noisy conversational settings (see e.g.,* *Geravanchizadeh et al., 2021). Currently AI platforms struggle to match human listeners and deliver unsatisfactory performance*.

**Comment 2.2:**

P5 lines 134-137
This paragraph consists of one sentence (which is not common) and it is difficult to understand. Please, split in two sentences.

**Response:** We simplified the sentence, and it now reads as follows:

Line 142–144:

*We used the accuracy of speech envelope reconstruction (SER) to study how selective attention and our other experimental manipulations affected the neuronal tracking of AV cocktail party speech****.***

The paragraph, where the sentence occurs is also now in the revised manuscript considerably longer.

**Comment 2.3:**
P10 Fig. 3
The font size is too small in several parts of the Fig (e.g."att", "ign", numbers)
This partly applies to Fig. 4 as well.

**Response:** We revised the font sizes in Fig. 3 and 4.

**Comment 2.4:**

P12, l 355
The link to [www.mv.helsinki.fi/home/jkaurama/vdialog/](http://www.mv.helsinki.fi/home/jkaurama/vdialog/) did not work for me (the other video links worked)

**Response:** We apologise the inconvenience. We have modified the link, and it should work now.

**Comment 2.5:**
P13, l 364
"there was initially correlations" should (probably) read as "there were initially correlations".

**Response:** Done as suggested

**Comment 2.6:**
P26, line 793
"the data was bandpass filtered (0.5-10 Hz)"
Please, add filter characteristics, if available.

**Response:** We used the MNE python raw.filter method, with a high pass cutoff of 0.5 and lowpass cutoff of 10, with the ‘firwin’ method and default parameters. This function computes the coefficients of a finite impulse response filter (hamming window). We now describe the method used for filtering in more detail in the revised text.

Line 850–855:

*After this, the formerly chosen deviant channels were interpolated, the data was bandpass filtered (0.5–10 Hz) using the mne.raw.filter function, with the ‘firwin’ option (default settings). This function computes the coefficients of a finite impulse response filter (hamming window). Thereafter the data were down sampled to 128 Hz for the temporal response function analyses and 64 Hz for the speech reconstruction analyses.*

**Comment 2.7:**
P29, line 867
"approximate effect sizes were applicable." should probably read as "approximate effect sizes where applicable."

**Response:** Done as suggested

**References added due to revision:**

49.	Rimmele JM, Golumbic EZ, Schröger E, Poeppel D. The effects of selective attention and speech acoustics on neural speech-tracking in a multi-talker scene. Cortex. 2015;68:144-54.

50.	Haider CL, Suess N, Hauswald A, Park H, Weisz N. Masking of the mouth area impairs reconstruction of acoustic speech features and higher-level segmentational features in the presence of a distractor speaker. Neuroimage. 2022;252:119044.

51.	Broderick MP, Anderson AJ, Lalor EC. Semantic Context Enhances the Early Auditory Encoding of Natural Speech. Journal of Neuroscience. 2019;39(38):7564-75. doi: 10.1523/Jneurosci.0584-19.2019. PubMed PMID: WOS:000486403700010.

60.	Kriegeskorte N, Kievit RA. Representational geometry: integrating cognition, computation, and the brain. Trends in cognitive sciences. 2013;17(8):401-12.

64.	Tóth B, Farkas D, Urbán G, Szalárdy O, Orosz G, Hunyadi L, et al. Attention and speech-processing related functional brain networks activated in a multi-speaker environment. PLoS One. 2019;14(2):e0212754.

73.	Large EW, Jones MR. The dynamics of attending: How people track time-varying events. Psychological review. 1999;106(1):119.

74.	Fritz JB, Elhilali M, Shamma SA. Differential dynamic plasticity of A1 receptive fields during multiple spectral tasks. The Journal of neuroscience. 2005;25(33):7623.

75.	Fritz JB, Elhilali M, Shamma SA. Adaptive changes in cortical receptive fields induced by attention to complex sounds. Journal of Neurophysiology. 2007;98(4). doi: 10.1152/jn.00552.2007. PubMed PMID: WOS:000250082700045.

76.	Reed A, Riley J, Carraway R, Carrasco A, Perez C, Jakkamsetti V, et al. Cortical map plasticity improves learning but is not necessary for improved performance. Neuron. 2011;70(1):121-31.

78.	Geravanchizadeh M, Roushan H. Dynamic selective auditory attention detection using RNN and reinforcement learning. Scientific Reports. 2021;11(1):15497.
